# Analysis of Taste Quality Differences Between High and Low Grades of Ninghong Tea: From the Perspective of Sensory, Metabolite, and Taste Activity Values

**DOI:** 10.3390/foods13233957

**Published:** 2024-12-08

**Authors:** Cuinan Yue, Zhihui Wang, Hua Peng, Lianghui Jiang, Puxiang Yang, Wenjin Li

**Affiliations:** 1 Jiangxi Cash Crops Research Institute, Nanchang 330043, China; m18306083532@163.com (C.Y.); ghost20066@126.com (H.P.); 13970985935@163.com (L.J.); puxiangy@163.com (P.Y.); 2 Jiangxi Provincial Key Laboratory of Plantation and High Valued Utilization of Specialty Fruit Tree and Tea, Nanchang 330043, China; 3 College of Agriculture, Jiangxi Agricultural University, Nanchang 330045, China; 4 College of Horticulture, Fujian Agriculture and Forestry University, Fuzhou 350002, China

**Keywords:** Ninghong tea, black tea, taste, metabolism, taste activity value

## Abstract

In this study, the taste quality difference between high (Ninghong-Jinhao tea, JH, unfolded fresh leaves) and low (Ninghong-Congou tea, CG, unfurled fresh leaves) grades of Ninghong tea (unique black tea) was analyzed from the perspective of sensory omics, non-targeted metabolomics, and chemical dose. JH was characterized by sweetness and mellowness with umami, while CG was characterized by sweetness and thickness. A total of 94 differential metabolites contribute to the quality difference between two grades. Further quantitative analysis revealed that JH exhibited a high accumulation of amino acids, catechins, and theaflavins, while CG demonstrated a high accumulation of water extract, tea polyphenols, flavonol glycosides, and saccharides. Taste activity values (TAVs) analysis revealed that the key taste components of JH and CG were catechin, epigallocatechin gallate, three theaflavins, caffeine, myrictin-3-*O*-glucoside, quercetin-3-*O*-rutinoside, quercetin-3-*O*-glucoside, quercetin-3-*O*-galactoside, kaempferol-3-*O*-rutinoside, kaempferol-3-*O*-glucoside, and gallic acid. Among the identified compounds, the TAVs of five flavonol glycosides in Ninghong tea were found to be greater than 10 for the first time. This study is helpful to understand the taste quality difference between different grades of Ninghong tea from the molecular sensory level, providing a scientific foundation for quality improvement and targeted regulation.

## 1. Introduction

Black tea, a fully fermented tea, represents the most produced and sold tea in the world, accounting for about 78% of the global tea trade [1]. Black tea is highly prized for its pleasant flavor and numerous purported health benefits. The quality of black tea is determined by a number of factors, including its appearance, color, taste, aroma, and leaf base. The taste is sensed by the cells of the degustator and flows through the body, so it is highly regarded [2]. The taste of tea is primarily derived from non-volatile substances, including phenols, amino acids, sugars, and organic acids. These factors affect the quality characteristics and nutritional value of a tea infusion [3]. The maturity of fresh leaves varies, resulting in differing accumulations of endogenous metabolites. Consequently, the quality of black tea processed from them differs. Consumers generally believe that the earlier and less ripe the tea is, the better the quality. Previous studies have also shown that the final quality of tea depends on the chemical composition of the fresh leaves, and different grades of fresh leaves have different metabolic compositions in the same type of tea, which can be processed into different grades of tea [4]. For example, catechin metabolism changes with the degree of cell aging, and the expression levels of *CsSCPL4* and *CsSCPL5*, the key enzyme genes of the ester catechin synthesis pathway, are significantly higher in buds than in leaves and stems, while the expression levels of *CsTA*, the key enzyme gene of the catabolic pathway, were higher in leaves with higher development maturity [5]. These studies showed that the quality components accumulated in different parts of the tender tea plant exhibited distinct profiles, which ultimately influenced the flavor quality of the finished tea.

In China, there are a wide range of black tea-producing areas, among which Congou black tea is the most common, and Ninghong tea is the earliest black tea variant in history [6]. Ninghong tea is divided into Ninghong-Jinhao tea (JH) and Ninghong-Congou tea (CG). The fresh leaf of the former is primarily composed of buds, while the latter is mainly buds and leaves with connected stems. The distinct compositions of these two types of Ninghong tea have their own characteristics, but also determine their economic benefits and affect the choices of consumers. However, at present, the quality difference between JH and CG has not been reported. Systematic analysis of the quality of the two grades of Ninghong tea is conducive to a clear understanding of their quality characteristics, which helps producers to target processing and consumers to make reasonable decisions. Metabolomics can be used to characterize small molecular weight compounds in biological samples, and it has been widely used to analyze the metabolic characteristics of foods [7,8,9]. Non-targeted metabolomics, based on ultra-high performance liquid chromatography-tandem mass spectrometry (UPLC-MS/MS), can be used to isolate and identify hundreds of small-molecule compounds in the same system [10,11]. In recent years, this technology has been widely applied to the analysis of endogenous metabolites in tea, with the objective of elucidating the influence of different tea cultivars, processing technologies, cultivation management strategies, and ecological environments on tea quality [12,13,14,15]. However, the sensory properties of tea do not correspond to the taste characteristics of metabolites one by one, but are formed by the addition, antagonism, and transformation of various components on taste buds [16]. Therefore, it is difficult to judge the flavor of tea only from the types of metabolites. Quantitative description analysis (QDA) is a food quality evaluation technique that quantitatively evaluates the flavor, appearance, and texture of food based on certain scales and relying on sensory organs. It is also used to evaluate the flavor of tea [17]. The development of chemometrics provides an effective method for the in-depth analysis and interpretation of instrument readings [18]. Taste activity value (TAV) refers to the ratio of the content of a certain flavor component to its taste threshold, and this method is effectively used to screen the key taste compounds in tea [19]. These technologies provide a convenient means of conducting this research.

In this study, QDA, non-targeted metabolome, phytochemical composition, and taste activity values were used for the first time to comprehensively analyze the taste quality differences between high and low grades of Ninghong tea, with the aim of clarifying the molecular mechanisms behind their taste quality differences. This study is helpful to understand the taste quality difference between different grades of Ninghong tea from the molecular sensory level, providing a scientific foundation for quality improvement and targeted regulation.

## 2. Materials and Methods

### 2.1. Samples and Chemicals

#### 2.1.1. Tea Samples

**Figure 1:**
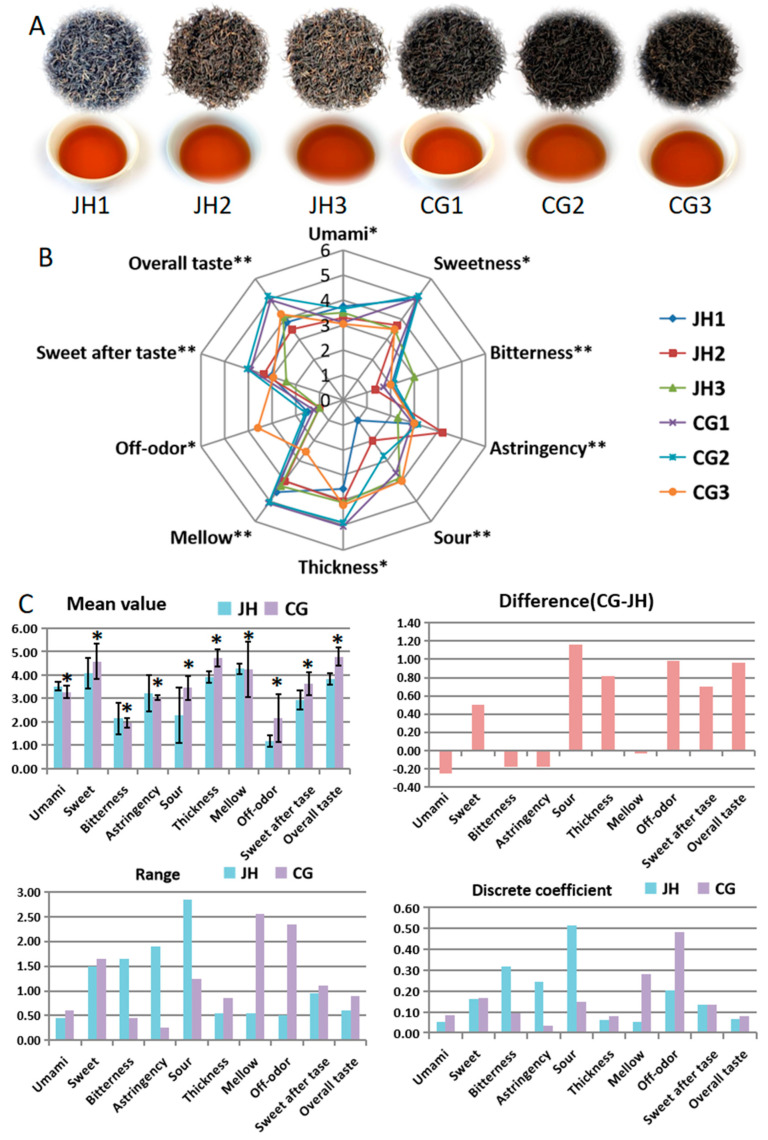
The sensory evaluation of Ninghong tea of two grades. (**A**) Images of dry tea and tea infusions of Ninghong tea of two grades. (**B**) The QDA of Ninghong tea of two grades. * *p* < 0.05, ** *p* < 0.01. (**C**) Difference and discretization analysis of JH and CG. * There is a significant difference between the two groups when *p* < 0.05. The black line represents the standard deviation of taste attributes for the same group of samples. JH, Ninghong-Jinhao; CG, Ninghong-Congou. JH1, JH2, and JH3 represent three samples of Ninghong-Jinhao, respectively. CG1, CG2, and CG3 represent three samples of Ninghong-Congou, respectively.

Representative samples were collected from representative tea enterprises in the local core production areas of two types of Ninghong tea, namely, 3 Ninghong-Jinhao (JH) and 3 Ninghong-Congou (CG). All samples were processed from fresh leaves of the Ningzhou population species according to the *Code of practice for processing of Xiushui Ninghong tea* (DB/36T 1629-2022, DB/36T: Local standard of the People’s Republic of China) [20]. The processing steps are withering, rolling, fermentation, and drying. The sample details are shown in Table 1. Dry tea and tea infusions are shown in Figure 1A.

#### 2.1.2. Chemicals

Catechin (C), epicatechin (EC), epicatechin gallate (ECG), epigallocatechin gallate (EGCG), catechin gallate, epigallocatechin (EGC), gallocatechin gallate (GCG), gallic acid (GA), and caffeine (CAF), were purchased from Prafa Technology Development Co., Ltd. (Chengdu, China), at a purity of 98% or higher. Theaflavin (TF), theaflavin-3-gallate (TF-3-G), theaflavin-3′-digallate (TF-3′-G), theaflavin-3,3′-digallate (TF-3,3′-diG), and rutin, were purchased from Yuanye Bio-Technology Co., Ltd. (Shanghai, China), at a purity of 98% or higher. Twelve standards such as quercetin, quercetin-3-*O*-rutinoside, quercetin-3-*O*-galactoside, quercetin-3-*O*-glucoside, kaempferol, myricetin, kaempferol-3-*O*-glucoside, kaempferol-3-*O*-rutinoside, kaempferol-3-*O*-galactoside, myricetin-3-*O*-rutinoside, myricetin-3-*O*-galactoside, and myricetin-3-*O*-glucoside were purchased from Aifa Biotechnology Co., Ltd. (Chengdu, China), at a purity of 98% or higher.

### 2.2. Quantitative Description Analysis

The QDA of the sample was performed as we reported previously [21]. Twelve staff were tested for the sensory evaluation roles. The tests included their ability to distinguish, evaluate, describe, and sense the taste. According to the GB/T 16291.1-2012 (GB/T: national standards of the People’s Republic of China) [22], solutions with different taste characteristics (sweetness: 16.0 g/L sucrose solution; sourness: 1.0 g/L citric acid solution; bitterness: 0.5 g/L caffeine solution; astringency: 0.5 g/L alum solution; saltiness: 5.0 g/L sodium chloride solution; metallic taste: 0.01 g/L hydrated ferrous sulfate solution) were prepared for selecting evaluators. The staff whose accuracy rate for the test results was not less than 80% would be regarded as qualified and serve as evaluators for the samples in this study. Finally, six evaluators (three males and three females, 20–60 years old) were selected to form an evaluation team.

Referring to the reported taste characteristics of black tea [23,24], a reference system for evaluating the taste characteristics of six Ninghong teas was developed in combination with the taste profiles of our sample (Table 2). The established taste reference system and non-experimental black tea samples were used to train evaluators, with emphasis on the use of descriptors and scale intensity labeling of 1–7 (1 = weakest, 4 = medium, and 7 = strongest). Each training lasted for 2 h, consisting of six sample evaluations 3–5 times a week until each evaluator could accurately, skillfully, and stably use descriptors and sensory scales before evaluating the test samples.

Three grams of sample were placed in a special cylindrical evaluation cup, added with 150 mL boiling water, covered, soaked for 5 min, and strained into the evaluation bowl to evaluate its taste sub-attributes and strength. The tea infusion was labeled by three-digit random coding to hide tea sample information, the processed tea infusion was assigned to evaluators to evaluate its taste, and the results were recorded. Each employee was evaluated independently without any discussion. Each evaluator performed six evaluations per round. Each sample was repeated three times. After each evaluation, the next sample was evaluated after a 5 min rest. Evaluation environment requirements were that it was clean and odorless, with a temperature of 20–25 °C. The average value of the evaluation results of the six evaluators was taken as the final result of the sample.

### 2.3. Non-Targeted Metabolomics Analysis

Twenty milligrams of the ground sample were put into a 2 mL centrifuge tube and 70% methanol aqueous solution containing 1 µg/mL 2-chlorophenylalanine was added, then vortexed for 3 min, given ultrasonic treatment in an ice water bath for 10 min, vortexed for 1 min, refrigerated at −20 °C for 30 min, and centrifuged at 4 °C and 13,400× *g* for 10 min. Subsequently, 300 μL of supernatant was removed into a new 2 mL centrifuge tube, centrifuged at 4 °C and 13,400 × *g* for 3 min, and 200 μL of supernatant was removed into the lining tube of the sample bottle for UPLC-MS/MS analysis. The experimental method and data processing of UPLC-MS/MS were consistent with the previously published literature [21]. The formula for calculating Fold change (FC) is as follows:

Fold change = Log2(CGCx/JHCx) (1)

CGCx is the response value of compound x of the sample mean value of CG; JHCx is the response value of compound x in the mean value of JH. Three biological replicates were used for each sample.

### 2.4. The Quantitative Analysis of Quality Components

The tea’s polyphenol content was determined according to GB/T 8313-2018 [25]. Gallic acid was used to make a standard curve. The amino acids content was determined according to GB/T 8314-2013 [26]. L-glutamate was used to make a standard curve. The water extract content was determined according to the weight loss differential method (GB/T 8305-2013) [27]. The saccharide content was determined by the Plant Soluble Sugars Assay Kit (Nanjing Jiancheng Biology Co., Ltd., Nanjing, China) according to the manufacturer’s instructions. Glucose was used to make a standard curve. Total flavones were determined by the AlCl_3_ method [21].

The CAF, GA, C, EC, EGC, catechin gallate, EGCG, ECG, GCG, TF, TF-3-G, TF-3′-G, and TF-3,3′-di-G were determined by 1260 High Performance Liquid Chromatography (HPLC) (Agilent, Santa Clara, CA, USA) according to Yue et al. [21]. The 250 mm × 4.6 mm × 5 µm Hypersil BDS C18 chromatographic column (Thermo Fisher Scientific, Waltham, MA, USA) was used. The 12 flavonols and their glycosides were determined by 1260 HPLC (Agilent, Santa Clara, CA, USA) according to Wang et al. [2]. The 250 mm×4.6 mm×5 µm ZORBAX ODS chromatographic column (Agilent, Santa Clara, CA, USA) was used. Three biological replicates were used for each sample.

### 2.5. Calculation of Taste Activity Value (TAV)

TAV is calculated as shown in the following formula:

$$\mathrm{TAV}=\frac{Cx}{R\times M}\times 10000$$

Cx is the concentration of taste compounds in mg/g; R is the recognition threshold in μmol/L; M is the relative molecular mass.

### 2.6. Data Statistics and Analysis

The one-way analysis of variance (ANOVA) with least significant difference (LSD) were performed by SPSS (version 19.0; Chicago, IL, USA). Principal component analysis (PCA), cluster analysis, and orthonormal partial least-squares discriminant analysis (OPLS-DA) were performed using SIMCA (version 14.1; Umetrics, Umea, Sweden). The heat map was drawn by Metware Cloud (https://cloud.metware.cn, accessed on 8 August 2024).

## 3. Results and Discussion

### 3.1. Taste Attributes of JH and CG and Their Differences

According to the evaluation procedure mentioned above, qualitative and quantitative evaluation was carried out on the taste attributes of two grades of Ninghong tea. The nine descriptors (Table 2) and overall flavor intensity were used to evaluate the sample, and the results are shown in Figure 1B,C. The sensory analysis showed that JH was characterized by sweetness and mellowness with umami, while CG was characterized by sweetness and thickness. These findings corroborate the notion that sweetness represents a defining attribute of Ninghong tea, aligning with the preceding analytical outcomes [21]. There were significant differences between JH and CG in the attributes of umami, sweetness, thickness, and odor, and in bitterness, astringency, mellowness, sweetness of aftertaste, and overall taste. However, not all of JH’s taste attributes differed significantly from CG’s. The thickness and overall taste strength of the CG group were higher than that of JH group; from this point of view, it suggested that low-grade Ninghong tea has better brewing value. CG had a higher acidity than JH. In the process of manufacturing Ninghong tea, the fermentation time of CG is longer than that of JH, which facilitates the accumulation of organic acids and promotes its stronger sour taste [28]. Through inter-group comparative analysis, it was found that JH had a higher umami, mellowness, bitterness, and astringency than CG, while its sweetness, sourness, thickness, off-odor, aftertaste, and overall intensity were lower than CG (Figure 1C). In addition, the highest taste intensity of JH was sweet (4.07) and the lowest was sour (1.0), while CG’s were thickness (4.72) and bitterness (1.95), respectively. This analysis confirmed the difference in taste between JH and CG.

The intra-group dispersion of two grades of Ninghong tea was analyzed by range and coefficient of dispersion (Figure 1C). The range of umami, sweetness, thickness, mellowness, off-odor, sweetness of aftertaste, and overall intensity of JH was lower than that of CG, while bitterness, astringency, and sourness were the opposite. The dispersion coefficients of JH and CG show the same trend in terms of range, except for sweetness of aftertaste. These two indexes can better reflect the dispersion of samples in the intra-group. This implied that the umami, sweetness, thickness, mellowness, off-odor and overall intensity of JH were more stable than CG, but the bitterness, astringency and sourness of CG fluctuated less. This result reminds us that, when processing Ninghong tea, we should consider how to reduce the fluctuation of JH or CG around some taste attributes, so as to improve the overall taste quality.

### 3.2. An Analysis of Nonvolatile Metabolites of JH and CG

**Figure 2:**
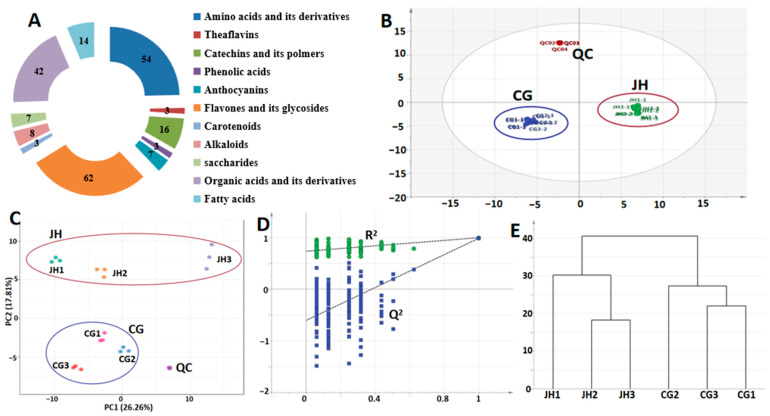
Nonvolatile metabolites of two grades of Ninghong tea. (**A**) The classification of metabolites. (**B**) The OPLS-DA model. R^2^X = 0.809, R^2^Y = 0.999, Q^2^ = 0.987. (**C**) The PCA of metabolites. R^2^X = 0.819, Q^2^ = 0.605. (**D**) Hypothesis testing of the OPLS-DA model. (**E**) A cluster analysis of the two grades of Ninghong tea. JH, Ninghong-Jinhao; CG, Ninghong-Congou; QC, quality control. JH1, JH2, and JH3 represent three samples of Ninghong-Jinhao, respectively. CG1, CG2, and CG3 represent three samples of Ninghong-Congou, respectively.

Non-targeted metabolomics analysis was performed on the metabolites of two grades of Ninghong tea, and a total of 219 quality-related metabolites were identified according to database comparison. The detailed information is shown in Table S1. The metabolites were classified into 11 categories, including 62 flavones and their glycosides, 54 amino acids and their derivatives, 42 organic acids and their derivatives, and 16 catechins and their derivative polymers, 14 fatty acids, 8 alkaloids, 7 anthocyanins, 7 saccharides, 3 theaflavins, 3 phenolic acids, and 3 carotenoids (Figure 2A).

PCA can reveal the similarities and differences between different samples through metabolites, and in the score distribution of the PCA model, samples that are close to each other show little difference in variables [16]. PCA was performed with all the identified quality metabolites as a variable matrix (Figure 2C). The four repetitions of QC samples could be effectively aggregated, indicating good repeatability and reliable data. The first 5 extracted principal components could explain 81.8% of the variable information, and the samples were all in the 95% confidence interval, indicating that the model had a high degree of explanation for the sample variables. In Figure 2C, JH and CG are separated by the first principal component (PC2 makes a positive contribution to JH and a negative contribution to CG), and neither of them crosses. However, there was a certain distance between the samples in the intra-JH and intra-CG. This indicated that the metabolites of the three JHs were similar, while the metabolites of the three CGs were similar. Additionally, some of the tastes were close among the intra-groups, but there were still large differences, which was consistent with the results of taste subattribute analysis (Figure 2C). Therefore, we further screened the differential metabolites of two grades of Ninghong tea.

### 3.3. Screening of Difference Metabolites of JH and CG

OPLS-DA is a “supervised” analysis method that “reduces dimension” of variables, establishes regression models, and performs discriminant analysis of regression results [17]. In order to identify the differential metabolites in JH and CG, OPLS-DA modeling and important variable projection analysis were carried out on the identified metabolites. It was found that JH and CG were situated in different areas in the model, exhibiting no overlap. This suggests that the model is capable of accurately differentiating between JH and CG (Figure 2B). In the score chart of the first two principal components, JH and CG are distinguished by the first principal component. The cross-validation showed that the model did not overfit (all blue Q^2^ points were lower from left to right than the original green R^2^ point, and the regression intercept of Q^2^ points is less than 0) (Figure 2D). The results of cluster analysis confirmed this conclusion. The JH group and the CG group were grouped into one cluster, respectively (Figure 2E). However, JH3 had a certain separation from other JHs, and CG1 was relatively far away from other CGs, which was consistent with the above PCA results. Hence, both OPLS-DA and clustering confirmed the difference between the metabolites of JH and CG.

**Figure 3:**
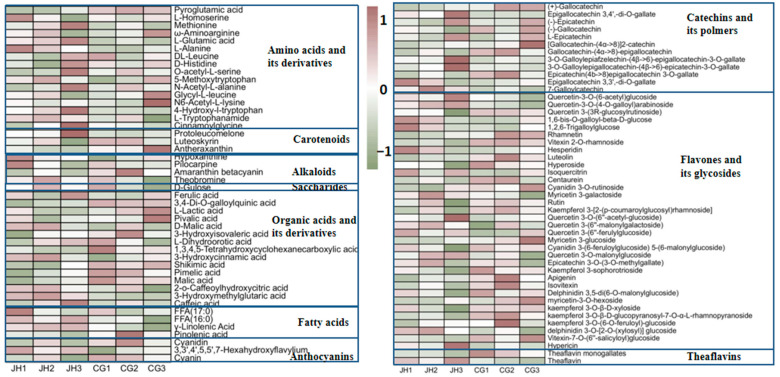
Heat map of the differential metabolites in JH and CG. Red on the same line indicates high content, while green indicates low content. JH, Ninghong-Jinhao; CG, Ninghong-Congou. JH1, JH2, and JH3 represent three samples of Ninghong-Jinhao, respectively. CG1, CG2, and CG3 represent three samples of Ninghong-Congou, respectively.

We screened differential metabolites according to the rule of VIP > 1.00 and *p* < 0.05, and identified 94 differential metabolites, including 34 flavones and their glycosides, 16 amino acids and their derivatives, 15 organic acids and their derivatives, 12 catechins and their polymers, 4 fatty acids, 4 alkaloids, 3 anthocyanins, 3 carotenoids, 2 theaflavins, and 1 saccharide. The heatmap was applied to visualize the relative variation in these differential metabolites among high and low grades of Ninghong tea (Figure 3). The fold change is shown in Table S2. Each column represents a sample, and each row represents a differential metabolite. A color-coded scale grading from green to purple corresponds to the content of the given metabolite shifting from low to high. The taste of tea is the comprehensive expression of metabolites in the mouth. Different compounds and the ratio of each compound determine the taste of tea, and the differences in tea taste are induced by the differences in compounds [23]. Therefore, we further analyzed the different compounds in the two grades of Ninghong tea.

**Amino acids and their derivatives**. Usually, amino acids are considered the source of tea’s umami, but, in fact, the tastes of amino acids are quite diverse. They can be divided into the following 4 types: (1) umami, including theanine, glutamic acid, glutamine, and aspartic acid; (2) sweetness, glycine, alanine, serine, ornithine threonine, and proline; (3) bitterness, isoleucine, leucine, valine, phenylalanine, tyrosine, and valine; (4) astringency, γ-aminobutyric acid [19]. Therefore, the difference in the type and content of amino acids also greatly affects the taste and quality of the tea. Among the 16 different metabolites of amino acids and their derivatives, 9 were up-regulated in JH, and the 3 most up-regulated were L-glutamic acid, 4-hydroxy-i-tryptophan, and methionine, which were higher than CG by 0.39-, 0.68-, and 0.53-fold, respectively. L-glutamic acid is an important contributor to umami taste, which may serve as a crucial foundation for the heightened umami perception in JH compared to CG. The umami intensity of JH was higher than CG (7.64%). In CG, the top three differential amino acids and their derivatives were pyroglutamic acid, N6-acetyl-L-lysine, and DL-leucine, by 1.75-, 0.5-, and 0.24-fold, respectively. Pyroglutamic acids are derivatives of glutamic acid, but have a sour taste, which provides the chemical basis for the strong sourness of CG. Under the condition of qualified tea quality, we think that umami intensity is high in unfermented tea (green tea) or semi-fermented tea (yellow tea, white tea, Oolong tea), and low in fermented tea (black tea and dark tea). In the previous sensory analysis, the umami intensity of JH and CG was lower than that of sweetness, mellowness and thickness. Additionally, high umami is also an important attribute of high-quality black tea. It has been reported that, the more tender the fresh leaves, the higher the amino acid content, but the amino acid in the tea stem is higher than that in the leaves. Therefore, the amino acid content in Ninghong tea can be increased through harvesting and processing, as one of the strategies to enhance its umami taste.

**Flavones and their glycosides.** Flavones, also called anthoxanthins, are a kind of yellow pigment widely existing in nature, their basic structure being 2-phenylchromone. Its C3 position is readily hydroxylated to form flavonols, and most of the flavones in tea combine with sugars to form flavone glycosides [29]. Flavone glycosides mainly cause dryness in the human mouth, with a velvety astringency. At the same time, they can also have a synergistic effect with bitter compounds, enhancing the bitterness of a tea infusion [16]. In two grades of Ninghong tea, a total of 34 differential flavones and their glycosides were screened, of which 14 were up-regulated in JH and 20 in CG. In JH, the up-regulation more than 0.5-fold of its flavones and glycosides was followed by quercetin-3-*O*-(4-*O*-galloyl) arabinoside, quercetin-3-*O*-(6-acetyl) glucoside, quercetin-3-*O*-(6-acetyl-glucoside), delphinidin-3-*O*-[2-*O*-(xylosyl)] glucoside, delphinidin-3-*O*-[2-*O*-(xylosyl)] glucoside, quercetin-3-*O*-malonylglucoside, isoquercitrin, and myricetin-3-*O*-galactoside, being up-regulated, respectively, 0.86-, 0.81-, 0.78-, 0.67-, 0.56-, 0.55-, and 0.52-fold. In CG, the flavones and their glycosides with more than 0.5-fold up-regulation were isovitexin, vitexin-2-O-rhamnoside, luteolin, apigenin, rhamnetin, vitexin-7-*O*-(6″-salicyloyl) glucoside, kaempferol-3-*O*-β-D-glucopyranosyl-7-*O*-α-L-rhamnopyranoside, cyanidin-3-*O*-rutinoside, cyanidin-3-(6-feruloylglucoside)-5-(6-malonylglucoside), delphinidin-3,5-di(6-*O*-malonylglucoside), myricetin-3-*O*-glucoside, and hyperoside. Their up-regulations were 1.07-, 0.96-, 0.89-, 0.82-, 0.81-, 0.81-, 0.69-, 0.62-, 0.62-, 0.56-, and 0.54-fold, respectively. In general, the up-regulated compounds in the two grades of Ninghong tea belonged to flavone glycosides, there were many up-regulated flavone glycosides in CG, and the up-regulated multiples were higher than those of JH. However, the sensory evaluation results indicated that CG was less astringent than JH (5.23%), which may suggest that flavone glycosides may not be the most important determinant of astringency of Ninghong tea. This finding is consistent with the conclusions of our previous study on the quality of 6 kinds of Congou black tea [21]. Therefore, we speculate that the astringency of Ninghong tea may be mainly provided by other astringency compounds, such as catechins. Zhang et al. [30] believed that most flavone glycosides were up-regulated more in low-grade Keemun black tea, but relatively less in high-grade Keemun black tea. This was similar to our conclusion. Flavones and their glycosides account for approximately 3–4% of the dry weight of tea. The concentration of these compounds is higher in spring tea than in summer tea, and lower in buds than in leaves [29]. This suggests that tea producers can harvest fresh leaves with low tenderness in summer and produce black tea with relatively high flavone glycosides to improve the taste strength of the tea and its potential health care effects.

**Catechins and their polymers.** Catechins are important providers of astringency and bitterness in tea [19]. During the processing of black tea, about 70% of catechins undergo oxidative polymerization, forming pigments or catechin complexes. These complexes contribute to the reduction in bitterness of the black tea, and they are also components of the steeping leaves of black tea [29]. A total of 12 differential compounds of catechins and their polymers were screened, among which 6 compounds were up-regulated in JH, including 7-galloylcatechin, 3-O-galloylepiafzelechin-(4β->6)-epigallocatechin-3-O-gallate, 3-O-galloylepigallocatechin-(4β->6)-epicatechin-3-O-gallate, epigallocatechin 3,4′,-di-O-gallate, epigallocatechin 3,3′,-di-O-gallate, and L-epicatechin; the up-folds were 1.57-, 0.61-, 0.42-, 0.38-, 0.34-, 0.07-fold, respectively. Up-regulated in CG were (+)-gallocatechin, [gallocatechin-(4α->8)]2-catechin, gallocatechin-(4α->8)-epigallocatechin, (−)-gallocatechin, epicatechin(4b->8)epigallocatechin-3-O-gallate, (−)-epicatechin; the up-folds were 0.90, 0.98, 0.80, 0.58, 0.32, 0.98, 0.98, 0.80, 0.58, 0.32, 0.90, 0.98, 0.80, 0.32, and 0.02, respectively. In JH and CG, the up-regulated differential compounds were mainly catechin dimers. 7-galloylcatechin was the most up-regulated catechin in JH. In our previous studies on the quality of Congou black tea in different regions, it was also found that the accumulation of 7-galloylcatechin in Ninghong tea was the highest (*p* < 0.05) [21]. Therefore, we hypothesized that 7-galloylcatechin may be the marker metabolite of Ninghong tea and be more abundant in buds than leaves. A limitation of this study is the lack of clarity regarding the flavor characteristics of 7-galloylcatechin. In addition, during the fermentation of black tea, catechins are oxidized to theaflavins or thearubigins, or to form diflavanols [29]. In addition, the oxidation process of buds is faster and stronger than that of leaves [31], and the fermentation time of JH is shorter than that of CG in the manufacturing process. Therefore, the degree of oxidation of catechins can be controlled by adjusting the proportion of bud and leaf or the degree of fermentation during the primary processing of Ninghong tea to achieve directional production.

**Organic acids and their derivatives.** Organic acid metabolism in plants is the result of biological oxidation, which is mainly accumulated through tricarboxylic acid metabolism and fermentation during breathing [32]. The organic acids in tea account for about 3% of dry tea, which is an important contributor to the sourness and the main reason for the acidity of tea infusions [23]. Previous studies have shown that the organic acid in black tea is higher than that in green tea, white tea, Oolong tea, and dark tea [33]. Sourness is regarded as a negative taste in tea infusion. In the sensory evaluation of tea, sourness is used as one of the indicators to judge the tea’s taste. If the tea infusion is too sour, it is thought to be due to over-fermentation or improper storage, leading to spoilage [32]. However, some scholars believe that the effect of sourness on tea infusion is double-sided; strong sourness is unfavorable to the tea taste, while weak sourness can improve the mellowness of tea and has a certain positive contribution to the umami taste of tea [23]. A total of 15 differential organic acids were detected in JH and CG. There were 7 up-regulates in JH, including 3-hydroxymethylglutaric acid, caffeic acid, 3-hydroxycinnamic acid, DL-malic acid, ferulic acid, 2-o-caffeoylhydroxycitric acid, and L-dihydroorotic acid, by 0.58-, 0.44-, 0.21-, 0.15-, 0.11-, 0.09-, and 0.04-fold, respectively. Up-regulated in CG were pimelic acid, 3, 4-di-o-Galloylquinic acid, shikimic acid, L-malic acid, 1, 5-tetrafluorobenzoic tetrahydroxycyclohexanecarboxylic acid, 3-hydroxyisovaleric acid, L-lactic acid, and pivalic acid, and up folds were 0.77, 0.47, 0.35, 0.25, 0.25, 0.24, 0.22, and 0.11, respectively. The accumulation of different organic acids in CG is greater. Studies have shown that the organic acids in fresh leaves decrease with the decrease in tenderness, but CG is higher in acids than JH, which may be caused by fermentation. The fermentation time of JH is 2.5–4.5 h, and CG’s is 3–6 h (DB36/T 1629-2022, as issued Jiangxi Provincial Market Supervision Administration) [20]. Long-term fermentation creates favorable conditions for the accumulation of organic acids in CG. The sour intensity of CG was higher than JH’s by 42.54%, which reflected the possible accumulation of more organic acids in CG. L-Malic acid has a higher sour intensity (stronger than citric acid), its sourness is longer lasting, and it is the main acid that affects the change in the total amount of organic acids, along with quinic acid and citric acid, in black tea [33], suggesting that the higher sour intensity in CG may be dominated by it. Malic acid is an intermediate product of TCA, and the long-term fermentation of black tea is also beneficial for the accumulation of malic acid. Therefore, when processing black tea, it is advisable to optimize the fermentation process parameters by regulating the metabolic indicators of organic acids.

**Theaflavins.** Theaflavins are a characteristic component in black tea, and their name is a generic term for compounds with a benzophenone structure formed by enzymatic oxidation and the condensation of a mixture of catechins [29]. Theaflavins exhibit a gentle astringency, but they form a complex with caffeine that has an umami taste. High theaflavins have also become synonymous with high-quality black tea, and their content is positively correlated with price [34]. Therefore, more and more researchers have focused on how to improve the theaflavins of black tea. There were two types of theaflavins with significant differences in JH and CG, among which theaflavin monogallates were up-regulated in CG and theaflavin was up-regulated in JH. Theaflavins are formed in three ways: (1) EC + EGC →→→→ TF1a; (2) EC+ (+) -GC →→→ TF1b; (3) (+) -C + EGC →→→ TF1c. The synthesis pathway of theaflavin monogallates is as follows: (1) EC + EGCG →→ TF-3-G; (2) (+) -C + EGCG →→ TF-3-G [29]. In the process of black tea fermentation, TFs and diflavanols can be coupled and oxidized to form thearubigins when EC, C, and ECG carriers are present. Therefore, the fermentation time lasts for a long time, which is conducive to the accumulation of thearubigins and the reduction in TFs. The fermentation time of CG is longer, which may lead to the decrease in TF. In our work, the total amount of theaflavins in JH was higher than that in CG. Owour et al. [35] reported that short-term fermentation is conducive to the accumulation of TF and TF-3′-G, and that TF-3-G and TF-3,3′-DG accumulate more with the extension of fermentation time. This is similar to our conclusion; that is, the amount of TF accumulated in JH with a relatively short fermentation time was higher, while the amount of TF-3-G accumulated in CG with a longer fermentation time was higher. It can be seen that, in production, the content of theaflavins in black tea can be regulated by adjusting the fermentation time, and then the value of black tea can be improved.

**Anthocyanins and carotenoids.** In tea infusion, anthocyanins provide astringency [36]. Carotenoids are fat-soluble pigments, which are the precursors of volatile aroma compounds such as terpenes [37]. There were 3 anthocyanins that were significantly different in JH and CG, among which 3,3′,4′,5,5′,7-hexahydroxyflavylium was up-regulated in JH, while cyanidin and cyanin were up-regulated in CG. This indicated that the accumulation of anthocyanins in leaves was higher than that in buds. Some studies have shown that black tea with high anthocyanin tea plant cultivars as raw materials has a special aroma and tastes better than conventional cultivars, which suggests that fresh leaves with low tenderness but high anthocyanin can be used to manufacture black tea with a special flavor.

**Alkaloids.** A positive correlation has been identified between the presence of common alkaloids and the bitterness of tea [23,29]. Four alkaloids were found to be significantly different in the JH and CG, namely hypoxanthine, theobromine, pilocarpine, and amaranthin betacyanin, the first three of which were up-regulated in JH and the latter in CG. The results of sensory evaluation showed that the bitterness of JH was higher than that of CG, while there was no significant difference in the bitterness of the intra-CG group, but the intra-JH group was the opposite, indicating that the bitterness intensity of Ninghong tea was not only dominated by caffeine (it is the key provider of bitterness in tea), but may result from other bitterness compounds.

**Saccharides.** Saccharides contribute to the sweetness and mellowness of black tea taste [2]. The saccharide that exhibits a notable discrepancy in the levels of JH and CG is D-gulose, which is up-regulated in JH. There are two biochemical processes related to enhancing the sweetness of a tea infusion during the processing of black tea: (1) the content of monosaccharides (fructose) is significantly increased, and the content of disaccharides is significantly decreased, and fructose is the saccharide with the highest sweetness intensity; (2) in the fermentation process of black tea, the enzyme induces the oxidation of catechins, which reduces the concentration of bitterness compounds [38]. These two paths are crucial to the formation of high sweetness and mellowness of a black tea. Therefore, in the process of black tea manufacturing, it is necessary to balance the accumulation of monosaccharides and the degree of catechin oxidation to improve the sweetness and mellowness of black tea.

**Fatty acids.** The influence of fatty acids on tea quality is mainly reflected in two aspects: (1) influence on the fullness of tea infusion; (2) it is an important aroma precursor [23]. Of the 4 differential fatty acids, FFA (17:0), FFA (16:0) and γ-linolenic acid were up-regulated in JH, while pinolenic acid was up-regulated in CG. In recent years, there have been more reports on the effect of fatty acids on the tea aroma [21], while there have been fewer reports on the effect of fatty acids on taste. Some studies have shown that during the storage of green tea, fatty acids undergo enzymatic oxidation and transform into odorous aldehydes, which make the tea deteriorate [39]. Hence, the influence mechanism of fatty acids on the taste of black tea and its changes during storage need to be further studied.

In general, the high and low grades of Ninghong tea have their own specific accumulation metabolites, which provide a biochemical basis for the identification and in-depth understanding of Ninghong tea grades. Furthermore, the combination of the absolute quantity of quality components can help researchers to more systematically understand the difference between the two grades of Ninghong tea.

### 3.4. Analysis on the Difference and Content of Main Quality Components in JH and CG

The absolute content, relative proportion, and interactive effects of various quality components in tea infusion act on taste receptors, forming the characteristic taste profiles of tea. In this part of the work, the absolute quantitative and inter-sample LSD analysis was performed on 29 indexes such as water extract content, total phenols, total free amino acid, caffeine, catechin monomer, theaflavin monomers, saccharides, theaflavins, and some flavonols and their glycosides in the samples (Table 3), so as to clarify the difference between the quality components of JH and CG. Among the 29 quality components, except quercetin, myricetin-3-*O*-glucoside, quercetin-3-*O*-rutinoside, and EGC, other indexes showed significant differences between JH and CG. Previous studies have shown that water extract has a great direct impact on the grade of black tea, and the content of water extract in tea reflects the concentration and strength of tea infusion. The water extract of JH was found to be lower than CG. There was no difference within the intra-JH group, but there was a significant difference in the intra-CG group, and CG2ʹs was significantly higher than that of CG1 and CG3. According to the results of sensory evaluation, the overall taste intensity of CG was higher than that of JH, among which CG2 was the strongest. However, significant differences were observed among the 6 samples, indicating that the taste intensity of Ninghong tea was not only regulated by the water leaching rate, but was closely related to other quality components.

The total phenols in the CG group were higher than that in JH group, but there were significant differences among samples in the intra-group, indicating that the phenols of the same grade of Ninghong tea from different manufacturers were also different, which we guessed might be affected by soil and cultivation management. The total flavones content in CG was higher than that in JH. Flavonols and their glycosides are the main flavones. Here, we studied the absolute quantification of 3 flavonols and 9 flavonol glycosides. Quercetin-3-*O*-glucoside, myricetin, and quercetin were more accumulated in JH, while other flavonols and flavonol glycosides were more accumulated in CG. Wu et al. [40] believed that the accumulation of flavonol glycosides in different parts of the tender shoots of tea plants was quite different; for example, the contents of kaempferol-3-*O*-galactoside and kaempferol-3-*O*-glucoside were the highest in the second leaf, and myricetin was the highest in the buds. There were more buds in JH and more leaves in CG, so their conclusion was basically consistent with ours. Flavonol glycosides have an additive effect on the bitterness of caffeine, and the strong thickness of black tea is related to the high level of flavonol glycosides [16]. Despite the high content of flavonol glycosides in CG, its astringency was lower than that of JH, indicating that it was not a decisive component of the astringency intensity of Ninghong tea. In the chemistry of black tea manufacture, the changes in catechin and gallic acid play a decisive role in the change in black tea taste quality [29]. Catechins are heterogeneous compounds with low bitterness and high astringency. Among these, non-ester catechins have mild astringency and light bitterness, while ester catechins show rough astringency and bitterness in the mouth [19]. In JH and CG, there were significant differences in catechins except EGC and catechingallate. Among them, the C, GCG, and ECG of JH were all higher than that of CG, and there was no significant difference between the three catechins in the intra-JH group, while there was a significant difference in the intra-CG group. The stability between samples in the intra-JH group was more stable, while the stability between samples in the intra-CG group was relatively poor. The content of EGCG and EC in CG was 4.16% and 5.58% higher than that in JH. EGCG and GCG were epimers of each other [29]. The accumulation of ester catechins in JH was more than CG, and the total catechins retained in JH were also more than CG, which may be the key reason why JH featured more bitterness and astringency than CG. Theaflavins are enzymatic oxidation products of catechins, which mainly result in astringency, and are considered to play a role in the umami and astringency of black tea. The results of our analysis indicated the presence of TF, TF-3-G, and TF-3,3′-di-G in JH and CG, which was in accordance with the findings of the metabolomic analysis, which did not identify TF-3′-G. Liu et al. [41] identified TF-3′-G in the study of metabolite changes during the processing of Ninghong tea. In JH and CG, the accumulation amount of three theaflavins was TF-3,3-di-G > TF-3-G > TF. The total amount of theaflavins, TF, and TF-3,3-di-G in JH were 4.7%, 11.6%, and 6.8% higher than those in CG, respectively. This is basically consistent with the previous metabolomics results. We suspected that this may be an important reason why JH was more astringent and umami than CG.

GA is a heterologous flavor compound that exhibits astringency, bitterness, and sourness in the mouth, and has a positive effect on the umami of green tea. The GA of JH was lower than that of CG, and there were significant differences within their respective intra-groups, which created conditions for the acid intensity of CG. In the process of black tea fermentation, GA easily reacts with C to convert theaflavic acid [29], but theanine acid was not identified by metabolomics, and we did not carry out absolute quantification of theanine acid.

In terms of saccharide content, JH was lower than CG, and there were significant differences in their respective intra-groups. The saccharides in tea contain monosaccharides, disaccharides, and some polysaccharides, and this work did not quantify the sugar monomers individually, therefore further analysis is needed.

Amino acid and caffeine are important nitrogen metabolites in tea plants, among which amino acid is an important substance for the umami and sweetness of tea infusions, and caffeine has a direct effect on the bitterness. The amino acid content of JH was higher than that of CG. There was no difference in amino acid content in the intra-JH group, but there was a significant difference in the intra-CG group. This provides the biochemical basis for JH’s umami advantage. The caffeine content of JH was 0.33% lower than that of CG, and only JH1 was significantly lower than the other 5 samples. Caffeine is an important bitter compound in tea infusion. In the analysis of differential metabolites, there was no significant difference between caffeine in JH and CG. Absolute quantitative results indicated that the caffeine level in JH1 was significantly lower than others. This suggests that caffeine is not responsible for the significant difference in bitterness between JH and CG. Xiao et al. [4] also confirmed that caffeine is not the key compound for the bitterness intensity of different grades of green tea. This is consistent with our view. Therefore, we assumed that there was no differential expression of caffeine in the tender shoot different parts (bud, first leaf, and second leaf) of the tea plant.

Overall, there were significant differences in the quality components between JH and CG. The contents of total free amino acids, total catechins, and total theaflavins in JH were higher than those in CG, while the contents of water extract, total phenols, total flavones, caffeine, GA, saccharides, and flavonol glycosides were lower than those in CG. The total amount of flavonols in both of them was the same, and there was no significant difference in quercetin content, while there was a significant difference in myricetin and kaempferol content between the two. The accumulation of differences in these quality components is a prerequisite for the difference in taste between JH and CG. However, the performance of quality components in the oral cavity is related to its threshold value. We also calculated the TAV of major taste compounds through the threshold value, so as to more comprehensively understand the reasons for the taste difference between JH and CG.

### 3.5. Difference Analysis of TAV of Main Quality Components in JH and CG

The threshold values of the 21 quality ingredients were obtained by searching the published literature, among which catechins included a bitterness threshold and an astringency threshold. Additionally, GA thresholds were divided into sourness, astringency, and bitterness. The TAV of the quality components was calculated using these thresholds (Table 4). The taste attributes of the 21 quality ingredients were divided into 5 categories, which are: (1) strong astringency and rough oral sensation; (2) puckering astringency and rough oral sensation; (3) mouth-drying and velvety-like astringency; (4) bitterness; (5) sourness. TAV greater than 1 indicates that the compound has a direct contribution to the tea’s taste and is a key taste substance [19]. The bitterness TAVs of 7 catechins was less than 1. The astringency TAV of EGCG was greater than 1 in all samples. The astringency TAV of C was greater than 1 in all samples except CG1. This manifested that EGCG and C were the key astringent compounds of Ninghong tea. The TAVs of the three theaflavins were all greater than 1 in JH and CG, among which TF-3,3′-di-G was the largest in JH2, and TF was the smallest inCG3. Previous studies have shown that theaflavin can be complexed with caffeine, and its complex has an important positive effect on the umami of tea [42]. This may be one of the reasons why JH has a higher umami taste than CG. Of the nine flavonol glycosides, the TAVs of myricetin-3-*O*-rutinoside, myricetin-3-*O*-galactoside, and kaempferol-3-*O*-galactoside were less than 1, and the others were all greater than 1. Quercetin-3-*O*-rutinoside has the largest TAV, followed by quercetin-3-*O*-galactoside, kaempferol-3-*O*-rutinoside, quercetin-3-*O*-glucoside, quercetin-3-*O*-glucoside, and quercetin-3-*O*-glucoside. Only myricetin-3-*O*-glucoside had a TAV less than 10. The TAV of quercetin-3-*O*-rutinoside and kaempferol-3-*O*-rutinoside in CG was greater than JH. The threshold of quercetin-3-*O*-rutinoside is extremely low, with TAVs reaching several thousand in JH and CG, which has a negative effect on the mellowness of black tea, which may be an important reason why the mellowness of CG was lower than JH. Previous studies have shown that the retention of flavonol glycosides in green tea is the largest, followed by white tea, and black tea is the lowest [16]. The reason for this is that the rolling and fermentation process of black tea promotes the hydrolysis and oxidation of flavonol glycosides, so black tea has the lowest flavonol glycosides. However, the TAV of flavonol glycosides still exceeded 1 in Ninghong tea, indicating that some flavonol glycosides still play a key role in the regulation of Ninghong tea taste. The three taste attributes of GA from low to high are astringency, bitterness, and sourness. Among JH and CG, only the astringency TAV exceeded 1, and it was higher in CG, suggesting that GA mainly provides astringency for Ninghong tea. The TAV of caffeine in JH was 4.04–4.58 and in CG was 4.23–4.34, indicating that caffeine was the key bearer of bitterness in JH and CG. In general, except for C, the key flavor compounds of JH and CG were similar, with only the TAV levels of each component being different.

In addition, the interaction between small molecule metabolites can also affect the strength of tea taste [21]. Flavonol glycosides can significantly enhance the bitter taste of caffeine. Conversely, caffeine has been demonstrated to accentuate the bitterness of catechin. Additionally, the combination of L-theanine and sucrose has been shown to markedly diminish the perception of bitterness associated with catechin [2,16,19]. The interactions between the key taste components of Ninghong tea may also affect its taste characteristics, and other components may also interact with the key taste components as cofactors to affect its taste characteristics. However, further investigation is required to elucidate the underlying molecular mechanisms governing the interaction between metabolites and their impact on the quality variation observed in Ninghong tea.

## 4. Conclusions

In conclusion, there were qualitative differences between JH and CG. JH had a dominant taste characteristic of sweetness and mellowness with umami, while CG was mainly sweet and thick. A total of 94 different metabolites contribute to the taste quality differences between the two. JH was highly enriched in amino acids, catechins, and theaflavins, while CG was highly enriched in water extract, tea polyphenols, flavonol glycosides, and saccharides. In addition, we found that C, EGCG, three theaflavins, caffeine, myrictin-3-*O*-glucoside, quercetin-3-*O*-rutinoside, quercetin-3-*O*-glucoside, quercetin-3-*O*-galactoside, kaempferol-3-*O*-rutinoside, kaempferol-3-*O*-glucoside, and GA were key taste components of Ninghong tea. In this study, the taste activity values of five flavonol glycosides in Ninghong tea were found to be greater than 10 for the first time, and these components may be the most critical contributors to its astringency. In the next step, we can further explore how to degrade five key flavonol glycosides in Ninghong tea, with the objective of enhancing its taste quality.

## Figures and Tables

**Table 1:** Detailed information of experimental samples.

Sample	Ninghong Tea Grade	Fresh Leaf Grade	Productive Time	Manufacturer
JH1	JH	Single bud, one bud, and one leaf at the beginning of development	April 2022	Jiangxi Wu’an Tea Industry Co., Ltd., Xiushui County, China
JH2			April 2022	Jiangxi Ninghong Co., Ltd., Xiushui County, China
JH3			April 2022	Jiangxi Dachun Tea Industry Co., Ltd., Xiushui County, China
CG1	CG	One bud, one leaf, and one bud, two leaves	April 2022	Jiangxi Dachun Tea Industry Co., Ltd., Xiushui County, China
CG2			April 2022	Jiangxi Ninghong Co., Ltd., Xiushui County, China
CG3			April 2022	Jiangxi Wu’an Tea Industry Co., Ltd., Xiushui County, China

JH, Ninghong-Jinhao; CG, Ninghong-Congou. JH1, JH2, and JH3 represent three samples of Ninghong-Jinhao, respectively. CG1, CG2, and CG3 represent three samples of Ninghong-Congou, respectively.

**Table 2:** Definition and reference of taste attributes of Ninghong tea.

Descriptors	Define	Reference Substance
Umami	Basic taste, the taste produced by glutamate or aspartate, the representative substance of which is glutamic acid.	Sodium glutamate aqueous solution
Sweetness	Basic taste, the response of sugars to taste organs.	Sucrose aqueous solution
Bitterness	Basic taste, taste of caffeine or quinine, etc., representing the substance for caffeine.	Caffeine aqueous solution
Astringency	A sense of traction, roughness, dryness, tension, and convergence in the mouth, tongue surface, and throat	KAl(SO_4_)_2_·12H_2_O aqueous solution
Sour	Basic taste, stimulating taste produced by substances such as malic acid, lactic acid, and citric acid.	Citric acid aqueous solution
Thickness	A sticky sensation. Tea infusion has a certain “elastic feeling” in the mouth.	Pectin solution
Mellow	The tea infusion in the oral cavity is relatively smooth and harmonious without any heterogeneity.	—
Off-odor	Including numb, rancid, aged, spicy, etc.	—
Sweet aftertaste	After drinking tea, there is a sweet sensation in the throat and tongue.	—
Overall taste	Comprehensive value of tea infusion taste.	—

**Table 3:** Content of taste quality compounds in JH and CG.

Compounds (mg/g)	JH1	JH2	JH3	CG1	CG2	CG3
Water extract	434.20 ± 3.93 a	430.22 ± 1.54 ab	426.15 ± 2.82 bc	420.84 ± 0.22 cd	436.99 ± 0.88 a	416.46 ± 3.45 d
Tea polyphenol	127.40 ± 0.15 d	135.56 ± 1.09 c	136.34 ± 1.46 b	134.63 ± 2.79 c	145.38 ± 1.00 a	137.73 ± 1.97 b
Total flavones	31.36 ± 0.57 b	32.05 ± 0.38 ab	31.33 ± 0.03 b	33.53 ± 0.71 a	32.41 ± 0.34 ab	31.73 ± 0.33 b
Amino acid	30.42 ± 0.41 b	28.74 ± 0.21 b	28.77 ± 0.50 b	23.51 ± 1.18 c	30.87 ± 0.31 a	24.16 ± 0.22 c
Caffeine	39.23 ± 0.55 b	40.25 ± 0.27 a	44.51 ± 1.59 a	42.12 ± 1.61 a	41.1 ± 0.54 a	41.18 ± 1.88 a
Gallic acid	6.01 ± 0.07 b	5.48 ± 0.04 c	5.94 ± 0.08 b	5.67 ± 0.01 c	5.55 ± 0.09 c	6.80 ± 0.08 a
Epigallocatechin	0.41 ± 0.00 a	0.37 ± 0.04 a	0.39 ± 0.01 a	0.51 ± 0.02 a	0.54 ± 0.13 a	0.50 ± 0.00 a
Catechin	6.03 ± 0.17 a	6.28 ± 0.00 a	6.13 ± 0.05 a	3.15 ± 0.02 d	4.84 ± 0.17 c	5.53 ± 0.15 b
Epicatechin	2.45 ± 0.03 c	3.55 ± 0.22 ab	4.20 ± 0.50 a	2.71 ± 0.09 bc	4.13 ± 0.43 a	3.39 ± 0.12 abc
Epigallocatechin gallate	5.30 ± 0.08 e	6.43 ± 0.05 c	7.23 ± 0.13 b	5.56 ± 0.09 e	8.24 ± 0.06 a	5.95 ± 0.06 d
Gallocatechin gallate	5.62 ± 0.39 a	5.22 ± 0.00 a	5.76 ± 0.03 a	2.08 ± 0.04 d	2.83 ± 0.13 c	3.84 ± 0.20 b
Epicatechin gallate	6.50 ± 0.08 a	6.78 ± 0.01 a	6.47 ± 0.07 a	3.39 ± 0.01 d	4.59 ± 0.12 c	5.46 ± 0.09 b
Catechingallate	2.45 ± 0.01 a	3.37 ± 0.01 a	2.54 ± 0.02 a	2.44 ± 0.01 a	2.49 ± 0.01 a	3.05 ± 0.70 a
Saccharides	21.89 ± 1.14 c	32.01 ± 1.11 b	19.03 ± 1.54 c	43.58 ± 2.01 a	32.74 ± 0.05 b	35.49 ± 3.03 b
Theaflavin	1.16 ± 0.02 a	1.11 ± 0.02 ab	0.99 ± 0.04 cd	1.05 ± 0.03 bc	0.96 ± 0.00 cd	0.91 ± 0.02 d
Theaflavin-3-gallate	2.40 ± 0.02 ab	2.54 ± 0.03 a	2.25 ± 0.09 bc	2.58 ± 0.06 a	2.55 ± 0.08 a	2.19 ± 0.03 c
Theaflavin-3,3′-digallate	4.09 ± 0.26 c	5.24 ± 0.16 a	4.27 ± 0.11 bc	4.73 ± 0.07 b	4.68 ± 0.04 b	3.33 ± 0.10 d
Myricetin-3-*O*-rutinoside	0.12 ± 0.03 ab	0.07 ± 0.00 b	0.07 ± 0.00 b	0.09 ± 0.01 ab	0.11 ± 0.01 ab	0.13 ± 0.00 a
Myricetin-3-*O*-galactoside	0.08 ± 0.01 b	0.10 ± 0.00 ab	0.10 ± 0.01 ab	0.11 ± 0.01 a	0.11 ± 0.00 a	0.11 ± 0.00 a
Myricetin-3-*O*-glucoside	0.11 ± 0.10 a	0.18 ± 0.00 a	0.19 ± 0.00 a	0.10 ± 0.09 a	0.26 ± 0.00 a	0.22 ± 0.00 a
Quercetin-3-*O*-rutinoside	0.11 ± 0.10 a	0.18 ± 0.00 a	0.19 ± 0.00 a	0.10 ± 0.09 a	0.26 ± 0.00 a	0.22 ± 0.00 a
Quercetin-3-*O*-glucoside	0.78 ± 0.01 a	0.59 ± 0.02 c	0.56 ± 0.00 c	0.48 ± 0.01 d	0.46 ± 0.01 d	0.71 ± 0.00 b
Quercetin-3-*O*-galactoside	2.10 ± 0.00 d	2.66 ± 0.06 b	1.97 ± 0.00 e	2.55 ± 0.01 c	2.88 ± 0.00 a	2.89 ± 0.01 a
Kaempferol-3-*O*-rutinoside	0.39 ± 0.01 d	0.45 ± 0.00 d	0.29 ± 0.00 e	0.72 ± 0.00 c	0.87 ± 0.01 a	0.76 ± 0.00 b
Kaempferol-3-*O*-galactoside	0.27 ± 0.00 b	0.23 ± 0.00 c	0.19 ± 0.00 e	0.22 ± 0.00 d	0.23 ± 0.00 c	0.29 ± 0.00 a
Kaempferol-3-*O*-glucoside	0.38 ± 0.01 d	0.64 ± 0.01 a	0.36 ± 0.00 e	0.49 ± 0.00 c	0.63 ± 0.00 a	0.51 ± 0.00 b
Myricetin	0.96 ± 0.00 b	0.90 ± 0.02 c	1.10 ± 0.01 a	0.95 ± 0.02 b	0.93 ± 0.00 bc	0.78 ± 0.00 d
Quercetin	0.95 ± 0.26 a	1.02 ± 0.03 a	1.04 ± 0.03 a	0.88 ± 0.03 a	0.81 ± 0.02 a	0.86 ± 0.00 a
Kaempferol	0.61 ± 0.04 ab	0.42 ± 0.03 b	0.95 ± 0.01 a	0.95 ± 0.03 a	0.79 ± 0.37 ab	0.99 ± 0.00 a

Note: Different letters in the same line indicate a significant difference at the *p* < 0.05 level. JH, Ninghong-Jinhao; CG, Ninghong-Congou. JH1, JH2, and JH3 represent three samples of Ninghong-Jinhao, respectively. CG1, CG2, and CG3 represent three samples of Ninghong-Congou, respectively.

**Table 4:** Thresholds of taste quality components and their TAV in the sample.

Compound	Threshold Value (μmol/L)	TAVs						Taste Characteristics
		JH1	JH2	JH3	CG1	CG2	CG3	
Epigallocatechin gallate	87	1.33	1.61	1.81	1.39	2.07	1.49	Strong astringency and rough oral sensation
Gallocatechin gallate	180	0.68	0.63	0.70	0.25	0.34	0.47	
Epicatechin gallate	160	0.92	0.82	0.91	0.48	0.65	0.77	
Catechingallate	250	0.22	0.30	0.23	0.22	0.23	0.28	
Epigallocatechin	160	0.08	0.08	0.08	0.10	0.11	0.10	Puckering astringency and rough oral sensation
Catechin	120	1.73	1.80	1.76	0.90	1.39	1.59	
Epicatechin	230	0.37	0.53	0.63	0.41	0.62	0.51	
Theaflavin	16	1.28	1.23	1.10	1.16	1.06	1.01	
Theaflavin-3-gallate	15	2.23	2.36	2.09	2.40	2.37	2.04	
Theaflavin-3,3′-digallate	13	3.62	4.64	3.78	4.19	4.14	2.95	
Myricetin-3-*O*-rutinoside	10.5	0.18	0.11	0.11	0.14	0.17	0.20	Mouth-drying and velvety-like astringency
Myricetin-3-*O*-galactoside	2.7	0.62	0.77	0.77	0.85	0.85	0.85	
Myricetin-3-*O*-glucoside	2.1	1.09	1.79	1.88	0.99	2.58	2.18	
Quercetin-3-*O*-rutinoside	0.00115	1567.67	2565.29	2707.80	1425.16	3705.41	3135.35	
Quercetin-3-*O*-glucoside	0.65	25.86	19.56	18.56	15.91	15.25	23.54	
Quercetin-3-*O*-galactoside	0.43	105.23	133.29	98.72	127.78	144.32	144.82	
Kaempferol-3-*O*-rutinoside	0.25	26.26	30.29	19.52	48.47	58.57	51.16	
Kaempferol-3-*O*-galactoside	6.7	0.90	0.77	0.63	0.73	0.77	0.97	
Kaempferol-3-*O*-glucoside	0.67	12.66	21.32	11.99	16.32	20.98	16.99	
Gallic acid	268.6	1.32	1.20	1.30	1.24	1.21	1.49	
Epigallocatechin	350	0.04	0.03	0.04	0.05	0.05	0.05	Bitterness
Catechin	290	0.72	0.75	0.73	0.37	0.57	0.66	
Epicatechin	230	0.37	0.53	0.63	0.41	0.62	0.51	
Epigallocatechin gallate	300	0.39	0.47	0.53	0.40	0.60	0.43	
Gallocatechin gallate	180	0.68	0.63	0.70	0.25	0.34	0.47	
Epicatechin gallate	350	0.42	0.37	0.42	0.22	0.30	0.35	
Caffeine	500	4.04	4.15	4.58	4.34	4.23	4.24	
Gallic acid	823	0.43	0.39	0.42	0.40	0.40	0.49	
Gallic acid	1099	0.32	0.29	0.32	0.30	0.30	0.36	Sourness

JH, Ninghong-Jinhao; CG, Ninghong-Congou; TAV, taste activity value. JH1, JH2, and JH3 represent three samples of Ninghong-Jinhao, respectively. CG1, CG2, and CG3 represent three samples of Ninghong-Congou, respectively.

## Data Availability

The original contributions presented in the study are included in the article/Supplementary Materials, further inquiries can be directed to the corresponding author.

## Supplementary Materials

The following supporting information can be downloaded at: https://www.mdpi.com/article/10.3390/foods13233957/s1. The data presented in this study are available within the manuscript and the Supplementary Materials. Table S1 Metabolite identification of all samples; Table S2 Detailed data on the differential multiples of metabolites.
