# Supplementary material for: Analysis of Taste Quality Differences Between High and Low Grades of Ninghong Tea: From the Perspective of Sensory, Metabolite, and Taste Activity Values
Source: Foods. 2024 Dec 8;13(23):3957. doi: 10.3390/foods13233957 (PMC11641024; doi:10.3390/foods13233957)
Supplement: Supplementary file 1 [file foods-13-03957-s001.zip › foods-3331640-supplementary.pdf]

Table S1 Metabolite identification of all samples

Compounds	Class	JH1	JH2	JH3	CG1	CG2	CG3
L-Theanine	Amino acids and its derivatives	2964,348	2681,911	2353,770	2075,667	3532,344	3053,031
Aminocaproic acid		7451	10013	12014	9123	10342	8210
DL-Glutamine		6724,068	7436,777	6725,572	6484,682	7620,323	7449,612
DL-Pyroglutamic acid		25077,075	38437,277	24653,074	33510,794	38565,872	38480,385
Asparagylasparagylarginine		36,665	13,762	9522	27,563	8522	27772
Pyroglutamic acid		20,074	17,081	42,098	79,560	93,454	82,321
Arginine		349,518	179,926	146,297	268,667	186,741	367,612
L-Isoleucine		4741,699	5260,691	7021,359	5303,721	6453,478	5551,092
L-Tryptophan		7188,411	7372,058	10146,309	7060,886	8224,474	7076,672
L-Threonine		203,891	183,811	153,209	184,705	173,165	189,659
L-Valine		6104,706	7268,104	11225,280	7893,586	8503,360	7344,640
5-Methoxytryptophol		3142	3600	4346	6327	4437	2844
L-Homoserine		101,755	86,284	66,267	86,207	76,872	88,633
Norvaline		535,445	297,218	217,899	481,788	275,597	394,276
Phenylalanine		61,458	60,077	57,628	58,035	48,705	85,176
Methionine		12,791	15,824	19,800	11,357	14,980	10,654
L-Ornithine		188,155	175,820	240,739	145,514	217,887	136,583
L-Leucine		23,311	15,887	66,162	15,332	18,914	19,182
L-Tyrosine		565,017	561,206	722,697	785,602	623,098	615,624
ω-Aminoarginine		16,921	18,275	13,649	15,251	15,864	19,512
L-Lysine		27,733	15,802	20,970	15,288	19,062	18,321
L-Glutamic acid		1001,683	1093,491	1329,600	674,643	813,155	648,921
Glutamine		82,218	57,550	31,128	58,690	45,781	54,599
L-Aspartic acid		278,669	296,527	366,211	378,836	253,023	244,055
L-Serine		141,750	130,854	149,465	165,788	131,608	137,864
L-Alanine		391,407	367,588	349,196	324,656	320,275	338,407
Prolintane		179	225	390	278	364	306
Citrulline		3342,102	3086,000	2466,894	2884,096	2993,221	3087,786
D-Ornithine		181,582	124,085	128,503	143,873	148,182	151,036
DL-Leucine		2216,080	1320,922	4018,057	4223,132	1616,736	3065,026
Ornithine		102,960	69,017	15,106	41,135	83,992	86,855
L-Homoarginine		60,238	56,878	44,160	67,951	42,876	25,499
Leu-Asn		49,591	47,432	42,037	47,601	54,813	48,794
β-Alanine		5448,343	5168,986	4195,485	4779,441	5011,651	5040,270
Tryptophan		14,839	3797	9889	8810	4769	42,599
DL-Proline		421,247	351,269	326,111	377,495	405,953	469,400
DL-Cysteine		6887	3206	4594	6215	3476	11763
DL-Arginine		1126,529	1298,053	1427,313	1134,894	1429,793	1176,401

D-Histidine		21,588	16,480	31,195	25,911	20,954	26,932
Acetylleucine		39,778	33,608	94,947	81,172	22,102	21,327
1-Methylpyrrolidine-2-carboxylic acid		3233,188	4580,652	2800,855	3773,902	4775,720	2356,969
N-Acetylvaline		113,959	185,311	283,949	183,549	244,313	158,809
O-acetyl-L-serine		161,629	170,597	249,573	186,205	219,407	185,408
N-methyl-L-glutamic Acid		193,420	304,210	364,468	275,037	375,688	320,652
5-Methoxytryptophan		1208,868	1426,216	1037,216	736,685	912,108	1392,545
N-Acetyl-L-aspartic acid		24,911	22,353	20,639	30,422	17,635	15,933
S-(5-Adenosy)-L-Homocysteine		95,547	91,279	48,252	72,654	88,464	97,474
N-Acetyl-L-alanine		319,371	356,068	510,176	358,840	446,738	372,828
Glycyl-L-leucine		166,830	175,588	135,170	151,521	160,093	196,929
N-(1-Oxoallyl)aspartic acid		585	394	1032	578	326	375
N6-Acetyl-L-lysine		7588	3985	5965	4085	2244	18446
Methionyl-Arginine		9959	6335	4126	5823	5673	9661
4-Hydroxy-l-tryptophan		155,233	178,337	226,214	132,473	132,137	123,833
L-Tryptophanamide		55,496	57,554	54,075	48,774	55,145	42,081
Cinnamoylglycine		18,757	24,654	35,644	25,349	23,414	20,193
Theaflavin monogallates	Theaflavins	121,260	121,669	102,594	188,315	163,941	143,756
Theaflavin		188,359	167,869	208,995	143,086	129,701	119,263
Theaflavin 3,3'-di-gallate		9649,049	13211,206	11434,859	11702,082	12492,480	8557,040
Epigallocatechin	Catechins and its polmers	922,072	1228,220	2428,178	1518,152	2588,674	2011,389
(+)-Gallocatechin		143,267	167,542	212,872	212,202	387,990	375,111
Gallocatechin gallate		844,714	957,386	1309,804	1042,558	1280,270	1243,747
(-)-Epicatechin		266,849	211,399	449,903	261,953	258,566	422,663
(-)-Gallocatechin		596,784	496,781	282,999	757,269	469,317	824,493
Epigallocatechin gallate		588,388	657,960	978,165	697,729	852,513	727,417
L-Epicatechin		171,656	142,629	272,285	132,410	177,172	250,872
Epigallocatechin 3,4',-di-O-gallate		184,955	172,260	232,159	149,931	151,738	149,737
[Gallocatechin-(4α->8)]2-catechin		565	891	1170	935	1446	2781
Gallocatechin-(4α->8)-epigallocatechin		229,958	419,742	208,588	535,544	609,654	347,174
3-O-Galloylepiafzelechin-(4β->6)-epigallocatechin-3-O-gallate		279,843	266,934	534,165	205,969	273,636	227,099
3-O-Galloylepigallocatechin-(4β->6)-epicatechin-3-O-gallate		325,014	384,762	619,557	345,172	344,945	306,610
Epicatechin(4b->8)epigallocatechin 3-O-gallate		170,548	197,733	172,409	230,461	250,847	192,665
Epigallocatechin-(4bβ->8)-epicatechin-3-O-gallate ester		60,995	73,151	181,169	110,338	99,484	165,993
Epigallocatechin 3,3',-di-O-gallate		58,922	46,936	26,166	30,086	33,476	40,976
7-Galloylcatechin		96,465	170,846	109,384	35,381	66,982	24,502
Chlorogenic Acid	Phenolic acids	301,766	191,450	401,052	202,022	315,278	574,259
Gallic acid		74325,25	70309,75	68844,14	70899,49	70763,62	79343,37

Theogallinin		8<br>1946,428	9<br>2726,793	6<br>2994,435	6<br>1952,971	1<br>2520,072	4<br>1953,822
Cyanidin	Anthocyanins	9028	16,359	8228	16,143	20,512	12,223
3,3',4',5,5',7-Hexahydroxyflavylium		3277,503	2763,098	3083,708	1793,499	2354,409	2508,576
Procyanidin		844	329	758	177	343	1262
Delphinidin 3,5,3'-triglucoside		2085	1543	1810	1709	1668	2537
Stevioside		129,822	143,551	129,862	125,286	147,064	143,766
Cyanin		326,822	549,559	187,753	652,559	608,319	425,246
Leucodelphidin		10,555	11,241	13,268	9346	8607	11,049
Quercetin-3-O-(6-acetyl)glucoside		178	130	258	145	117	60
Quercetin-3-O-(4-O-galloyl)arabinoside		1184	1421	1547	886	817	581
Quercetin-3-O-(2-malonyl)sophoroside-7-O-arabinoside		309	674	1407	806	914	275
Quercetin 3-(3R-glucosylrutinoside)		625,447	642,560	445,011	714,902	712,088	675,496
6-Chlorocatechin		97,186	72,150	139,811	109,006	93,343	126,785
1,6-bis-O-galloyl-β-D-glucose		594,618	520,434	361,188	337,132	346,178	468,897
1,2,6-Trigalloylglucose		309,493	248,650	171,132	175,653	157,427	214,727
Rhamnetin		131,290	157,944	195,848	201,108	340,780	311,237
Apigenin 7-(6''-malonylglucoside)		12,307	9806	39,046	17,261	17,046	31,541
Naringenin		576,119	621,943	886,766	701,522	653,945	693,612
Vitexin 2-O-rhamnoside		133,498	147,242	100,960	254,366	264,677	223,450
Cyanidin 3-rutinoside-5-glucoside		34,597	25,480	34,636	13,388	27,449	37,140
Isoginkgetin		40,550	17,782	57,532	31,795	29,774	130,266
Myricetin	Flavones and its glycosides	376,088	277,838	513,656	356,881	375,560	40,3502
Peonidin 3-arabinoside		63,444	96,822	165,401	66,199	101,948	96,851
Hesperidin		7497	7065	6226	6298	6432	6715
Luteolin		173,906	229,464	217,572	343,060	472,801	337,814
Hyperoside		1134,555	1116,514	551,898	1623,000	1263,703	1186,893
Isoquercitrin		349,129	191,594	326,807	168,622	154,794	270,267
Taxifolin		114,151	330,645	249,790	264,256	380,561	190,580
Centaurein		370,763	523,423	338,157	651,653	680,121	390,150
Cyanidin 3-O-rutinoside		20,070	15,665	35,136	36,871	29,721	47,364
Myricetin 3-galactoside		246,109	310,924	254,294	219,940	208,497	136,033
Rutin		2061,921	2835,975	1086,479	2570,336	2835,621	2462,542
Narirutin		279,433	161,862	253,569	162,720	198,579	345,358
Cyanidin 3-O-rutinoside 5-O-β-D-glucoside		715	325	845	437	865	1014
Kaempferol 3-[2-(p-coumaroylglucosyl)rhamnoside]		21,473	32,526	17,985	33,586	37,198	30,471
Epicatechin 3-glucoside		38,530	119,732	55,326	102,391	115,935	78,242
Naringin		27,989	15,733	22,619	22,570	15,507	26,244
Quercetin		1132	1776	1575	1777	1661	3315

3-O-(3',6-O-di-p-coumaroyl)-glucoside						
Delphinidin 3-O-(6''-O-malonyl)-β-glucoside	21,604	16,176	16,438	17,248	15,520	20,520
Quercetin 3-O-(6''-acetyl-glucoside)	37,850	28,249	85,023	19,680	30,758	37,830
Quercetin 3-(6''-malonylgalactoside)	739,937	784,666	712,350	572,175	764,189	657,578
Quercetin 3-(6''-ferulylglucoside)	3100,050	2268,457	2828,465	1748,465	1861,817	2846,309
Naringin 6''-rhamnoside	6063	8189	5897	6145	8547	7450
Myricetin 3-glucoside	857,884	829,353	1025,167	1004,484	1277,131	1709,459
Cyanidin 3-O-(6''-acetyl-arabinoside)	35,940	23,873	28,668	26,609	27,730	35,347
Cyanidin 3-(6-feruloylglucoside) 5-(6-malonylglucoside)	42,637	28,802	7598	45,337	36,313	40,034
Cyanidin 3-(6''-ferulylglucoside) 5-glucoside	2163	939	1476	1603	1631	3654
Apigenin 7-[glucuronyl-(1->2)-glucuronide] 4'-glucuronide	51,406	48,282	37,352	38,821	38,237	44,942
Quercetin 3-O-malonylglucoside	14,743	20,977	21,938	13,231	17,505	8490
Epicatechin 3-O-(3-O-methylgallate)	6310	6663	5624	6310	6406	5546
Kaempferol 3-sophorotrioside	7198	6432	5583	8182	7137	7305
Peonidin 3-sophoroside 5-glucoside	1124	2244	1329	2565	2220	1793
Quercetin-3-D-xyloside	1959	939	1570	1845	2264	2038
Naringenin-7-O-glucoside	10,364	13,512	20,377	12,381	21,564	18,225
Apigenin	13,719	22,103	20,823	30,788	43,079	25,817
Kaempferol	595,004	723,853	495,733	540,268	601,826	551,770
Isovitexin	354,861	680,498	209,317	829,500	1219,197	556,663
Prodelphinidin A2 3'-gallate	12,268	21,205	18,591	21,696	46,297	9765
Isorhamnetin-3-O-rutinoside-4'-O-glucoside	1299	761	994	418	675	895
Delphinidin 3,5-di(6-O-malonylglucoside)	3313	2193	666	4114	2215	3156
oleanolate 3-O-beta-D-glucoside	3086	2188	3268	2338	4075	4422
myricetin-3-O-hexoside	237,919	237,226	301,342	251,023	337,949	385,508
kaempferol 3-O-β-D-xyloside	210,290	175,451	144,568	208,463	207,236	211,585
kaempferol 3-O-β-D-glucopyranosyl-7-O-α-L-rhamnopyranoside	163,336	181,462	131,422	259,691	291,227	215,471
kaempferol 3-O-(6-O-feruloyl)-glucoside	653	976	1000	1028	1613	828
delphinidin 3-O-[2-O-(xylosyl)] glucoside	45,366	50,280	35,704	26,069	36,945	19,556
delphinidin 3-O-(6-O-malyl-β-D-glucoside)	1246	1898	1986	1941	2330	1011
cyanidin 3,5-di-O-beta-D-glucoside	2609	2774	1337	2365	2758	2763

Vitexin-7-O-(6"-salicyloyl)glucoside		2328	2094	1772	3834	2606	4435
Hypericin		266,990	249,467	310,055	234,310	254,478	240,880
Protoleucomelone	Carotenoids	4327	4018	7534	3245	2984	3475
Luteoskyrin		26,473	28,942	21,903	30,559	30,468	25,687
Antheraxanthin		12,161	10,243	9838	13,599	13,842	19,237
Hypoxanthine	Alkaloids	99,795	79,377	74,609	49,399	66,145	82,585
Pilocarpine		23,094	19,484	17,016	21,175	18,641	18,604
Arecoline		146,918	84,816	72,504	130,033	78,295	181,832
Caffeine		46234,257	45513,402	47551,913	47321,751	44796,933	44133,199
Etofylline		16,219	6307	2967	10,234	4598	34,870
Amaranthin betacyanin		1434	3377	1447	4302	6657	3507
Pentoxifylline		3418	4032	9375	4138	4663	3062
Theobromine		20034,085	20881,800	19657,417	20519,228	19333,788	18258,164
Lactobionic acid	saccharides	1960	1703	3792	3967	2233	1517
β-D-Galactose		375,016	860,246	903,009	930,691	815,609	879,852
β-D-allose		241,886	268,810	242,080	241,094	276,767	233,737
D-Erythrose		4584,555	4519,207	4437,872	4877,289	3889,367	3951,171
D-Fructose		261,601	228,735	92,702	205,551	184,576	212,711
D-Galactose		2461,074	2606,902	2473,919	2479,248	2307,539	2195,066
D-Gulose		90,881	100,745	91,881	102,265	84,050	75,080
Limonoic acid	Organic acids and its derivatives	21,216	27,171	14,566	23,582	24,229	25,480
Salicylic acid		94,285	97,589	134,335	98,552	117,044	116,595
4-Pentenoic acid		38,938	36,385	14,490	41,944	38,720	28,955
Ferulic acid		16,497	13,172	18,146	14,004	13,591	16,573
3,4-Di-O-galloylquinic acid		65,305	74,181	76,428	102,096	98,749	98,593
L-Lactic acid		1529,308	1273,369	1266,071	1367,864	1599,186	1764,894
Propionic acid		408,870	165,678	99,403	176,860	118,713	440,008
Citric acid		33476,723	34449,586	32220,450	29808,428	36582,807	34160,904
Pivalic acid		86,940	72,674	80,069	81,762	79,525	96,694
Mesylate		700	414	1095	848	328	726
DL-Malic acid		8761,202	8947,941	7472,994	7036,484	8585,736	7054,504
3-Hydroxyisovaleric acid		167,907	217,639	223,325	234,732	273,482	213,137
L-Dihydroorotic acid		1415,355	1720,429	1807,626	1879,425	1590,714	1354,096
1,3,4,5-Tetrahydroxycyclohexanecarboxylic acid		11937,322	11122,937	11112,385	14804,807	12767,077	13015,676
Lactic acid		18,501	21,496	13,805	19,707	18,275	14,487
Betulonic acid		287	1020	751	139	866	153
Threonic acid		3201,265	2239,030	1519,404	2425,876	1853,254	3082,252
Isocitric acid		16458,592	18298,836	17018,550	15961,521	18926,536	17406,867
3-Hydroxybutyric acid		242,893	158,410	177,911	141,857	148,089	276,381

3-Hydroxycinnamic acid		690,260	720,050	584,794	503,086	586,324	639,375
3-Methyl-2-oxovaleric acid		62,956	16,3615	95,209	123,264	155,769	116,859
Cinnamic acid		124,412	81,626	97,839	98,030	79,161	117,026
Shikimic acid		913,064	871,543	1082,637	1248,563	1164,449	1248,610
Pimelic acid		74,879	121,336	152,252	231,259	215,264	149,663
Glutaric acid		9032,153	8393,690	5649,820	6888,110	8605,239	7423,134
(2E,4E)-2,4-Hexadienoic acid		540,381	490,829	336,901	449,447	442,095	410,739
Maleic acid		1027,541	1087,974	871,898	842,875	1028,229	867,170
Succinic acid		4959,924	4714,488	3668,292	4080,100	4587,643	4501,056
3,4-Dihydroxyhydrocinnamic acid		171,778	144,692	191,605	168,974	180,519	187,125
Valproic acid		26,391	21,189	23,370	25,799	26,169	27,683
Cafestol acetate		47,909	46,554	46,740	46,479	47,571	48,103
L-Malic acid		65,405	71,024	74,245	88,439	81,702	80,998
2-o-Caffeoylhydroxycitric acid		43,353	43,887	41,025	39,955	43,232	37,321
2-O-Feruloylhydroxycitric acid		37,763	40,229	44,803	37,519	44,008	44,450
Chorismic acid		23,055	23,910	26,341	26,231	24,993	27,374
3-Hydroxymethylglutaric acid		41,742	44,262	37,889	29,091	26,653	26,872
Caffeic acid		31,010	56,939	79,529	33,854	55,305	34,277
Crotonic acid		151,491	125,301	89,346	122,173	116,507	127,855
2-Oxoarginine		327	367	807	960	448	425
Isoferulic acid		175,994	182,784	201,891	128,899	125,272	210,439
Dihydrocaffeic acid 3-O-glucuronide		1845	2060	4126	3774	2638	1893
4-Amino-3-phenylbutanoic acid		33,346	29,245	50,675	30,283	20,151	81,636
FFA(9:0)		384,420	365,934	314,790	351,149	346,753	333,688
FFA(18:1)		1670,753	1596,961	1635,268	1664,409	1621,668	1635,185
FFA(17:0)		59187,694	54038,493	52754,426	56676,002	52187,621	53774,157
FFA(16:1)		583,366	516,768	629,739	619,213	552,739	572,775
FFA(16:0)		1672,787	1635,795	1685,465	1599,776	1612,995	1533,469
FFA(14:0)		273,009	323,776	269,767	280,932	302,636	264,670
FFA(12:0)	Fatty acids	129,033	116,420	107,738	121,397	110,784	134,481
FFA(11:0)		175,898	163,944	163,119	168,371	156,544	160,295
γ-Linolenic Acid		10053,015	10120,551	10133,524	9997,794	9872,227	9752,683
11-Methyldodecanoic acid		52,884	34,176	100,315	60,742	40,222	68,359
Linoleic acid		28,738	22,248	24,955	17,405	15,574	27,615
Arachidic acid		16,825	12,776	11,428	14,786	14,602	18,364
Isovaleric acid		1717,277	2056,729	1388,402	1670,800	2024,760	1566,796
Pinolenic acid		6436	6183	3811	8273	14,074	8446

Note: The mean of three biological replicates.

Table S2 Detailed data on the differential multiples of metabolites

Index	Class	FC(JH/CG)	FC(CG/JH)
Pyroglutamic acid	Amino acids and its derivatives	−1.75	1.75
L-Homoserine		0.01	−0.01
Methionine		0.39	−0.39
ω-Aminoarginine		−0.05	0.05
L-Glutamic acid		0.68	−0.68
L-Alanine		0.17	−0.17
DL-Leucine		−0.24	0.24
D-Histidine		−0.09	0.09
O-acetyl-L-serine		−0.02	0.02
5-Methoxytryptophan		0.27	−0.27
N-Acetyl-L-alanine		0.01	−0.01
Glycyl-L-leucine		−0.09	0.09
N6-Acetyl-L-lysine		−0.50	0.50
4-Hydroxy-l-tryptophan		0.53	−0.53
L-Tryptophanamide		0.19	−0.19
Cinnamoylglycine		0.20	−0.20
Theaflavin monogallates	Theaflavins	−0.52	0.52
Theaflavin		0.53	−0.53
(+)-Gallocatechin	Catechins and its polmers	−0.90	0.90
Epigallocatechin 3,4',-di-O-gallate		0.38	−0.38
(-)-Epicatechin		−0.02	0.02
(-)-Gallocatechin		−0.58	0.58
L-Epicatechin		0.07	−0.07
[Gallocatechin-(4α->8)]2-catechin		−0.98	0.98
Gallocatechin-(4α->8)-epigallocatechin		−0.80	0.80
3-O-Galloylepiafzelechin-(4β->6)-epigallocatechin-3-O-gallate		0.61	−0.61
3-O-Galloylepigallocatechin-(4β->6)-epicatechin-3-O-gallate		0.42	−0.42
Epicatechin(4b->8)epigallocatechin 3-O-gallate		−0.32	0.32
Epigallocatechin 3,3',-di-O-gallate		0.34	−0.34
7-Galloylcatechin		1.57	−1.57
Cyanidin	Anthocyanins	−0.54	0.54
3,3',4',5,5',7-Hexahydroxyflavylium		0.45	−0.45
Cyanin		−0.66	0.66
Quercetin-3-O-(6-acetyl)glucoside	Flavones and its glycosides	0.81	−0.81
Quercetin-3-O-(4-O-galloyl)arabinoside		0.86	−0.86
Quercetin 3-(3R-glucosylrutinoside)		−0.30	0.30
1,6-bis-O-galloyl-β-D-glucose		0.36	−0.36
1,2,6-Trigalloylglucose		0.41	−0.41
Rhamnetin		−0.81	0.81
Vitexin 2-O-rhamnoside		−0.96	0.96
Hesperidin		0.10	−0.10
Luteolin		−0.89	0.89

Hyperoside		−0.54	0.54
Isoquercitrin		0.55	−0.55
Centaurein		−0.48	0.48
Cyanidin 3-O-rutinoside		−0.69	0.69
Myricetin 3-galactoside		0.52	−0.52
Rutin		−0.39	0.39
Kaempferol 3-[2-(p-coumaroylglucosyl)rhamnoside]		−0.49	0.49
Quercetin 3-O-(6''-acetyl-glucoside)		0.78	−0.78
Quercetin 3-(6''-malonylgalactoside)		0.17	−0.17
Quercetin 3-(6''-ferulylglucoside)		0.34	−0.34
Myricetin 3-glucoside		−0.56	0.56
Cyanidin 3-(6-feruloylglucoside) 5-(6-malonylglucoside)		−0.62	0.62
Quercetin 3-O-malonylglucoside		0.56	−0.56
Epicatechin 3-O-(3-O-methylgallate)		0.03	−0.03
Kaempferol 3-sophorotrioside		−0.24	0.24
Apigenin		−0.82	0.82
Isovitexin		−1.07	1.07
Delphinidin 3,5-di(6-O-malonylglucoside)		−0.62	0.62
myricetin-3-O-hexoside		−0.33	0.33
kaempferol 3-O-β-D-xyloside		−0.24	0.24
kaempferol 3-O-β-D-glucopyranosyl-7-O-α-L-rhamnopyranoside		−0.69	0.69
kaempferol 3-O-(6-O-feruloyl)-glucoside		−0.40	0.40
Delphinidin 3-O-[2-O-(xylosyl)] glucoside		0.67	−0.67
Vitexin-7-O-(6''-salicyloyl)glucoside		−0.81	0.81
Hypericin		0.18	−0.18
Protoleucomelone		0.71	−0.71
Luteoskyrin	Carotenoids	−0.17	0.17
Antheraxanthin		−0.53	0.53
Hypoxanthine		0.36	−0.36
Pilocarpine		0.03	−0.03
Amaranthin betacyanin	Alkaloids	−1.21	1.21
Theobromine		0.06	−0.06
D-Gulose	saccharides	0.12	−0.12
Ferulic acid		0.11	−0.11
3,4-Di-O-galloylquinic acid		−0.47	0.47
L-Lactic acid		−0.22	0.22
Pivalic acid		−0.11	0.11
DL-Malic acid	Organic acids	0.15	−0.15
3-Hydroxyisovaleric acid	and its	−0.24	0.24
L-Dihydroorotic acid	derivatives	0.04	−0.04
1,3,4,5-Tetrahydroxycyclohexanecarboxylic acid		−0.25	0.25
3-Hydroxycinnamic acid		0.21	−0.21
Shikimic acid		−0.35	0.35
Pimelic acid		−0.77	0.77

L-Malic acid		−0.25	0.25
2-o-Caffeoylhydroxycitric acid		0.09	−0.09
3-Hydroxymethylglutaric acid		0.58	−0.58
Caffeic acid		0.44	−0.44
FFA(17:0)	Fatty acids	0.03	−0.03
FFA(16:0)		0.07	−0.07
γ-Linolenic Acid		0.03	−0.03
Pinolenic acid		−0.91	0.91
